# Certain Dangers of Adenoid Operations

**Published:** 1913-10

**Authors:** 


Certain Dangers of Adenoid Operations. *Bulletin of the Johns Hopkins Hospital,* April, 1913. E. W. Grove, Milwaukee, Wis. The writer reviews the literature and his own experience for the complications and sequelæ of adenoid operations, such as meningitis sinusitis, mastoiditis and hæmorrhage, with the following recommendations: Delay operation when local or general contagion threatens. Take the child to a hospital when practical. Try to have a clean field of operation by preparatory treatment. Get the whole growth. The atlas forms a shelf in the posterior pharynx when the head is over-extended, behind which parts of the more luxuriant growths escape the curette, unless the head be slightly flexed. Have cases return for observation. The operation because of our great familiarity with it has bred all too much contempt.

